# Microbiome‐driven anticancer therapy: A step forward from natural products

**DOI:** 10.1002/mlf2.12118

**Published:** 2024-05-27

**Authors:** Yunxuan Guan, Di Wu, Hui Wang, Ning‐Ning Liu

**Affiliations:** ^1^ State Key Laboratory of Systems Medicine for Cancer, Center for Single‐Cell Omics, School of Public Health Shanghai Jiao Tong University School of Medicine Shanghai China

**Keywords:** anticancer, cancer, chemotherapy, immunotherapy, microbiota

## Abstract

Human microbiomes, considered as a new emerging and enabling cancer hallmark, are increasingly recognized as critical effectors in cancer development and progression. Manipulation of microbiome revitalizing anticancer therapy from natural products shows promise toward improving cancer outcomes. Herein, we summarize our current understanding of the human microbiome‐driven molecular mechanisms impacting cancer progression and anticancer therapy. We highlight the potential translational and clinical implications of natural products for cancer prevention and treatment by developing targeted therapeutic strategies as adjuvants for chemotherapy and immunotherapy against tumorigenesis. The challenges and opportunities for future investigations using modulation of the microbiome for cancer treatment are further discussed in this review.

## INTRODUCTION

The human microbiome inhabiting the human body consists of bacteria, fungi, archaea, and viruses^1^. Microbiota play a critical role in the maintenance of physiological activity and progression of diseases^2^, ^3^. Numerous studies have suggested a close relationship between microbiota and cancer^4^. The impacts of gut microbiota on tumor biology, including transformation processes, tumor growth, and immunotherapy response, have been demonstrated^5^. Interestingly, recent studies have demonstrated the existence, metabolic activity, and functional significance of intratumoral microbiota. However, numerous studies on host–microbiota interactions during tumor progression focus mainly on the gut microbiota^6^. In particular, microbiome dysbiosis can lead to a variety of pathogenic situations, ranging from gastrointestinal disorders, metabolic and cardiovascular diseases, to cancer^7^. Generally, gut microbiota contribute to tumorigenesis through several mechanisms: DNA damage, epigenetic regulation, inflammatory responses, and influence on antitumor immune responses.

Naturally derived chemical components contain a significant amount of bioactive substances with medicinal potential^8^. There is growing evidence that natural products and gut microbiota interact with each other bidirectionally^9^. By altering the pH in the host gastrointestinal tract, mucosal barrier integrity, and short‐chain fatty acid (SCFA) concentration, natural products can either prevent or promote microbial growth, leading to the alteration of the components, abundance, and metabolism of the gut microbiota^10^, ^11^. Therefore, exploring the interaction between natural products and gut microbiota will help us better understand the complicated mechanisms of natural products and their therapeutic effects.

The prevention and treatment of cancer can significantly benefit from targeting microbiota^12^. Natural products have shown potential in treating tumors by modulating the microbiota to regulate host pro‐inflammatory immune responses. Multiple investigations have unveiled the close connection between the efficacy of cancer chemotherapy and commensal microbiota. Therefore, natural products show promise in mitigating the side effects of chemotherapy and could serve as chemotherapy adjuvants^13^, ^14^. Natural products can function as immunomodulators, collaborating synergistically with immune checkpoint inhibitors (ICIs) to boost the efficacy of immunotherapy by, for instance, increasing the infiltration of cytotoxic T cells^15^. This review provides an overview of current knowledge about microbiota and cancer and outlines the molecular mechanism by which natural products interact with gut microbes. We further highlight the potential roles of natural products in anticancer therapy when combined with chemotherapy or immunotherapy mediated by microbiota. This review will provide new insights into the natural product–microbiome–cancer relationship and suggest ways to improve the clinical management of cancer patients.

## MICROBIOTA AND CANCER: THE CAUSALITY AND MOLECULAR MECHANISMS

**Figure 1:**
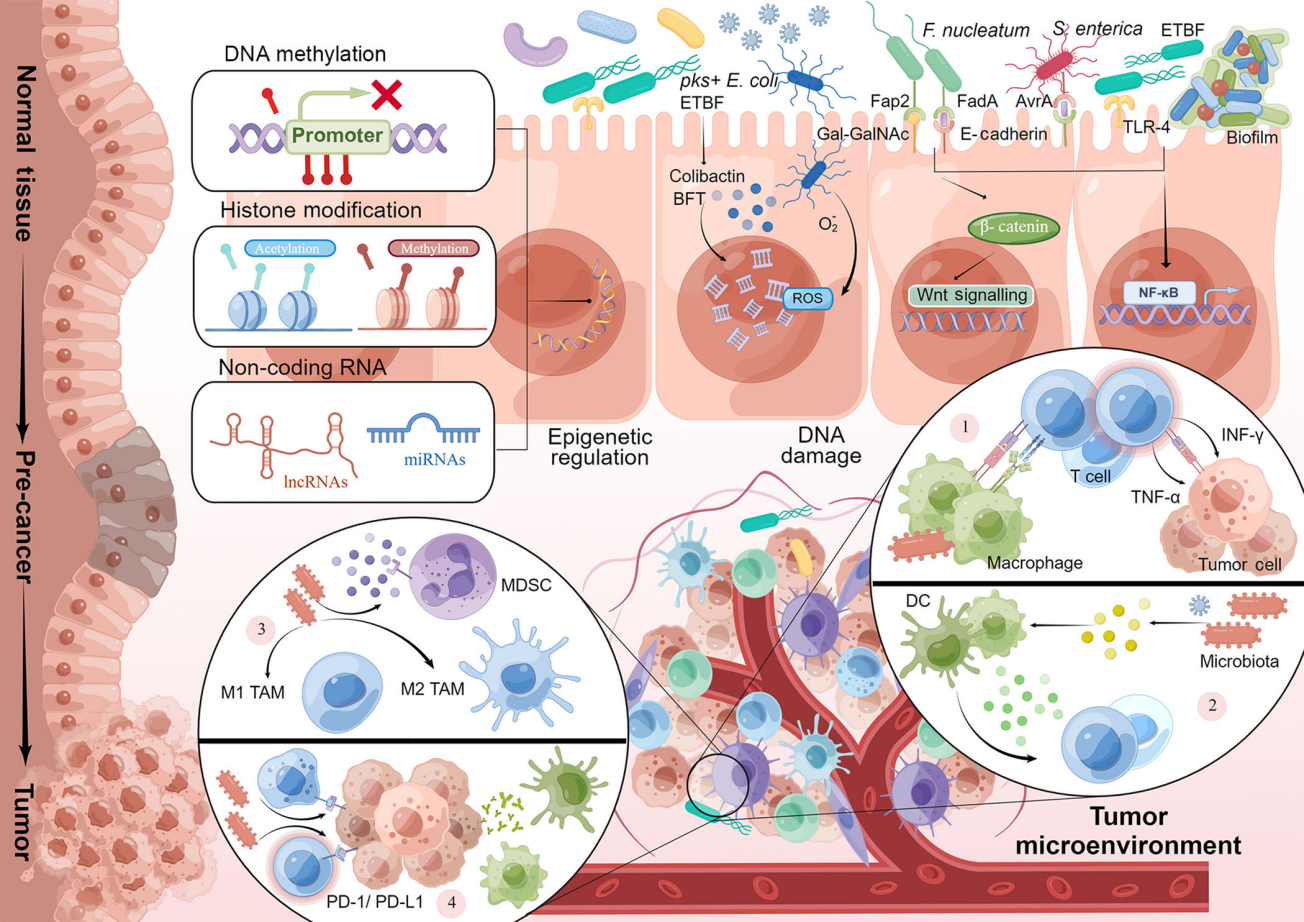
Interplay between microbiota and cancer through multiple mechanisms. The molecular mechanisms between microorganisms and tumors include direct DNA‐damaging effects of bacterial toxins and epigenetic regulation, inflammatory responses driven by signaling pathways following infection, and influence on antitumor immune responses. The microbiome plays an ambivalent role in antitumor immunity. (1) Microbial antigens could be presented by tumor cells, activating anti‐tumor immune responses. (2) The microbes stimulate inflammatory TIME by antigens and metabolites. (3) The microbes induce the differentiation of TAMs and recruit MDSCs, forming an immunosuppressive TME. (4) The microbes interact with inhibitory immune checkpoints, suppressing immune cells. BFT, *Bacteroides fragilysin*; DC, dendritic cell, ETBF, enterotoxigenic *Bacteroides fragilis*; MDSC, myeloid‐derived suppressor cell; ROS, reactive oxygen species; TAM, tumor‐associated macrophage; TIME, tumor's immune microenvironment; TLR‐4, Toll‐like receptor 4.

The human microbiota is a diverse and complicated ecosystem composed of symbiotic and parasitic microorganisms, known as “the Second Genome”. It is increasingly recognized that commensal microbiota play a critical role in different pathophysiological states, including precancerous lesions and oncogenesis. Besides the “oncogenic microbes”, the direct carcinogens recognized by the International Association for Cancer Registries, microbiota dysbiosis is demonstrated to initiate or promote cancer progression. The disturbance of microbiota (97% of which colonizes in the gastrointestinal tract, and others in the oral, pharyngeal, respiratory tract, skin, and genitourinary tract) contributes to cancer development via numerous community‐driven mechanisms^16^. The intratumoral microbiota might induce a distinct response by engaging in crosstalk with immune cells and tumor cells to impact the tumor microenvironment (TME)^17^. In addition, metabolites (i.e., butyrate) and secretes (i.e., colibactin) derived from microbiota also provide an exciting perspective to rethink cancer development^18^, ^19^. Collectively, the current molecular mechanisms between microbiome and tumor mainly involve genetic toxicity, epigenetic modification, signaling transduction, inflammation, and immune regulation (Figure 1).

### Genotoxicity and epigenetic regulation in microbial carcinogenesis

The microbiome has been reported to play a role in genetic or epigenetic regulation during cancer development. Colibactin produced by *pks* ^+^ *Escherichia coli* was found to cause DNA damage by inducing specific base mutations, DNA double‐strand breaks, and intrastrand DNA cross‐linking in vitro. The damage to DNA fostered tumors in a murine model of colorectal cancer (CRC)^20^. Furthermore, urinary tract infection, a risk factor for bladder cancer, is associated with DNA‐damaging *pks* ^*+*^ *E. coli* ^21^. These results suggest that *pks* ^*+*^ *E. coli* alters host genetics and facilitates tumorigenesis.

Regarding epigenetic regulation, DNA methylation induced by microbiota exposure has been observed both in mice models and in large CRC cohorts^22^, ^23^. Histone modification, including acetylation and methylation, has been commonly associated with microbial regulation via enzymes (i.e., histone methyltransferases vs. demethylases; histone acetyltransferase vs. deacetylases). It has been demonstrated that most histone modifications stem from SCFAs. Furthermore, the interplay between microorganisms and noncoding RNAs has been implicated in initiating epigenetic regulation^24^. *Fusobacterium nucleatum* modulates the expression of micro‐RNAs (miRNAs) and long‐non‐coding RNAs (lncRNAs) in the CRC mice model^25^, ^26^.

It has also been shown that *F. nucleatum* disrupts m^6^A modification in CRC cells^27^. Reciprocally, miRNAs secreted by host cells can upregulate bacterial gene transcription, forming a microbiota–host feedback loop via noncoding RNAs^28^. Collectively, improved meta‐genomics and multiomics technologies have unveiled the influence of specific microbial enrichment and microbial derivatives on the genetic code and expression, linking genetic predisposition to microbial dysbiosis with tumor progression. However, further investigation into the mechanisms of microbial‐mediated epigenetic modifications on the path to cancer (i.e., succinylation, lactylation, and lysine crotonylation) is still lacking. The relationship between microbial‐mediated epigenetic regulation, metabolic reprogramming, and immune regulation requires in‐depth investigation.

### Chronic inflammation and subsequent carcinogenicity

Protumorigenic inflammation promotes cancer development by initiating inflammatory signaling pathways, upregulating inflammatory mediators, and reshaping the TME to trigger inflammatory responses in epithelial cells^29^, ^30^. Inflammation‐related signaling pathways, such as the Toll‐like receptor (TLR) pathway, the nuclear factor κB (NF‐κB) pathway, and the Wnt/β‐catenin pathway, are closely associated with tumor development^31^, ^32^, ^33^ (Figure 1). An example is the association between gastric adenocarcinoma and chronic inflammation induced by *Helicobacter pylori*, driven through NF‐κB signaling pathway^34^. *F. nucleatum* uses virulence factor amyloid‐like *F. nucleatum* adhesin A (FadA) to bind to E‐cadherin to activate both the Wnt/β‐catenin and NF‐κB signaling pathways^35^, ^36^, ^37^. In colorectal and pancreatic cancers, NF‐κB can stimulate the generation of cytokines, including interleukin‐6 (IL‐6) and tumor necrosis factor, thus facilitating tumor progression and metastasis^38^, ^39^. The inflammatory environment promotes cell proliferation, inhibits apoptosis, and stimulates angiogenesis, all of which are characteristics of cancer cells.

Furthermore, microbiota‐induced inflammatory responses upregulate the DNA methylation levels of cancer‐related genes and increase the generation of reactive oxygen species (ROS), promoting inflammation‐driven carcinogenesis dependent on microbiota–genome crosstalk^40^, ^41^. However, apart from the limitations associated with individual variations and microbial diversity, the specificity of microbiota‐mediated inflammatory pathways poses a current challenge, and more in‐depth investigations are awaited to develop targeted drugs and minimize the overall impact on organisms.

### Microbial promotion and disruption of immune response

The TME is the driving force for tumor progression. It is highly heterogeneous, consisting of microorganisms, innate immune cells (i.e., neutrophils, macrophages, dendritic cells [DCs], mast cells, and natural killer cells [NK]), adaptive immune cells (i.e., T lymphocytes and B lymphocytes), and cancer cells^42^, ^43^. The tumor microbiome plays a significant and ambivalent role in antitumor immunity in two ways.

First, the microbiome can evoke adaptive antitumor immune responses through various mechanisms via microbial antigens. Tumor cells can recognize and engulf microbes, leading to the presentation of microbial antigen–major histocompatibility complexes, as well as pathogen‐associated molecule patterns, damage‐associated molecule patterns, and tumor‐associated antigens generated after cell lysis, further activating immune responses^44^, ^45^. Microbes share similar antigen epitopes with tumor cells can also promote immune responses through cross‐reactivity. This can effectively aid antitumor immunity that identifies or designs microbial peptides and “mimetic antigens” recognized by the immune system^46^, ^47^, ^48^. However, precise immune pathways targeting tumor cells instead of normal cells require further research and validation.

Second, microbes play a bidirectional regulatory role in the competition between tumor cells and the immune system. Intratumoral microbes induce the differentiation of M2‐like tumor‐associated macrophages (TAMs) and recruit myeloid‐derived suppressor cells (MDSCs), forming an immunosuppressive TME (Figure 1). For instance, pathogenic bacteria, including *Clostridium perfringens, Anaerobes*, and *Klebsiella pneumoniae*, can induce infiltration of M2‐like TAMs, thereby promoting the growth and metastasis of CRC^49^, ^50^, ^51^. In the presence of dysbiosis of the intestinal microbiota or pathogenic bacteria (such as *F. nucleatum*), CXCL1, a myeloid‐derived inhibitory cell chemoattractant that originates from CRC, can also be induced to promote intratumoral MDSC infiltration and suppress natural killer cells to evade immune destruction^49^, ^52^, ^53^, ^54^. The TLR–calmodulin–NFAT–IL‐6 signaling cascade of polymorphonuclear MDSCs is activated by microbiota, further facilitating microbial crosstalk with CRC cells, inducing tumor cells to express STAT3‐dependent coinhibitory molecules B7H3 and B7H4, and suppressing CD8^+^ T cell‐dependent antitumor immunity^55^. Additionally, microbes interact with inhibitory immune checkpoints, including PD‐1, CTLA‐4, TIM‐3, LAG‐3, TIGIT, and CEACAM1, suppressing the function of immune cells^56^, ^57^. A further explanation of the mechanisms governing the interaction between microbes and immune checkpoints may provide insights into the development of antitumor adjuvants.

## INTERACTIONS BETWEEN MICROBIOTA AND NATURAL PRODUCTS

Another primary mechanism through which microbes regulate cancer involves their metabolic byproducts. A new concept of “Pharmaco‐microbiome” describes the interplay between microbiota and drug responses^58^, ^59^. Similarly, the interaction between natural products and microbiota is manifested in both the microbial metabolism of natural products and the impact of natural products on the composition and functionality of the microbiota. The gut microbiota can metabolize these natural products, and the diverse metabolic pathways used by different microbial communities contribute to individual variability. Metabolites generated by gut microbiota can reach other parts of the body through systemic circulation, leading to distant effects via indirect interactions.

Additionally, natural products can modulate the abundance, composition, and activity of the gut microbiota, further shaping the interplay between the gut and the host. The primary mechanisms include the gut–liver axis, gut–brain axis, and gut–kidney axis^60^, ^61^. Natural products in the gastrointestinal tract, especially in the colon, readily interact with microorganisms due to their complex composition and prolonged retention. Hence, the interaction between natural products and microbiota offers new avenues for cancer therapy^62^.

### Transformation of natural products by gut microbiota

Gut microbiota can transform natural products to produce more active metabolites. For instance, bacteria ferment indigestible sugars, producing SCFAs crucial in cancer prevention and improvement^63^. Metabolites such as bile acids and adenosine can enter the bloodstream and modulate the tumor immune microenvironment (TIME)^64^. Another microbiota‐derived metabolite, trimethylamine‐*N*‐oxide (TMAO), has been found to enhance antitumor immune responses in triple‐negative breast cancer to date^65^. They act as signaling molecules, energy, and nutritional resources that may significantly impact the host's physiological activities, such as energy metabolism and immunological homeostasis. Gut microbiota possess metabolic capabilities akin to the liver due to their diverse enzymatic repertoire and robust metabolic activity^66^. Natural products are metabolized by the gut microbiota through diverse processes, including hydrolysis, oxidation, methylation, demethylation, and deamination^67^, ^68^. Through enzymatic hydrolysis induced by the gut microbiota, the biological functionality and bioavailability of certain large, poorly absorbable natural compounds are significantly improve^66^.

Methylation and demethylation processes play a pivotal role in the metabolism of the gut microbiota, enhancing the biological functionality of natural compounds^67^, ^69^. For example, the complete metabolization of 5,7‐DMF and 5,7,4′‐TMF produced 5,7‐dihydroxyflavone (chrysin) and 5,7,4′‐trihydroxyflavone (apigenin), which have potent anti‐cancer actions and efficacy in preventing a variety of chronic disease^70^. Natural products can also be transformed by oxidoreductases expressed by intestinal microorganisms. For instance, human intestinal flora can oxidize and reduce Oat avenanthramide‐C (2c), a unique polyphenol in oats, to the more active metabolite dihydroavenanthramide‐C (DH‐2c), which shows higher biological activity than 2c in inhibiting the development and inducing apoptosis in colon cancer cells^71^.

### Natural products mediated interference of in microbiome composition

Natural products can alter the composition of gut microbiota, modulating the balance between beneficial and harmful microorganisms, leading to dysbiosis, or aiding in restoring a healthy microbial community. For example, in mice with ulcerative colitis and CRC induced by Azoxymethane‐Dextran sulfate sodium (AOM/DSS), *Ganoderma lucidum* polysaccharide (GLP) treatment significantly promotes the growth of *Lactobacillus* and *Bifidobacterium*. GLP can also reduce the relative abundance of *Oscillibacter, Desulfovibrio, Alistipes, Lachnoclostridium*, and *Parasutterella*, some of which have been shown to be positively associated with CRC carcinogenesis^72^, ^73^. GLP effectively improved intestinal microbiota dysbiosis and reduced inflammation in AOM/DSS mice, which is expected to be used as a probiotic for the treatment of CRC^74^.

In addition, different gut microbiota show varying metabolic responses to natural compounds, and these natural compounds can modulate the composition of the gut microbiota in turn. The intricate interplay between them contributes to the occurrence and treatment of diseases. However, many studies exploring the correlation between gut microbiota and tumors show certain shortcomings related to natural compounds. First, the effects of microbiota‐derived metabolites have shown contradictory results in different mouse models. Second, most studies focus on the biological functions of gut microbiota‐derived metabolites, while neglecting the regulatory effects and mechanisms of dietary components, drugs, and other compounds on gut microbiota. Finally, few algorithmic models exclude the influence of natural compounds as confounding factors or integrate them with gut microbiota as covariates. Therefore, the action mechanisms of active metabolites require further investigation before adoption into clinical practice.

## DEVELOPMENT OF COMBINATORIAL ANTICANCER THERAPEUTICS WITH NATURAL PRODUCTS

Antitumor therapy is transitioning from the traditional oncology model to a multidisciplinary teamwork (MDT). First, microbial composition varies significantly among individual patients, different tumor subtypes, or even in the same patient at different tumor stages. Therefore, precisely identifying the microbial composition within an individual has significant guiding implications for drug screening and precision therapy. For example, the gut mycobiome plays a role in predicting the efficacy of ICIs, with higher predictive accuracy when considering both fungal and bacterial profiles^75^. It was demonstrated that intratumoral microbiome signature (*Pseudoxanthomonas–Streptomyces–Saccharopolyspora–Bacillus clausii*) efficiently predicted long‐term survival in pancreatic adenocarcinoma patients^76^. Additionally, the goal of comprehensive treatment is to leverage the distinct mechanisms of various treatment modalities, such as chemotherapy, immunotherapy, and targeted therapy, to enhance therapeutic outcomes. Regarding this, intervention strategies targeting commensal microbiota to improve chemotherapeutic efficacy, overcome resistance, and alleviate toxicity provide potential for clinical translation. For instance, chemotherapeutic drugs damage both tumor cells and normal cells due to “indiscriminate attacks”. Natural products modifying microbiota could improve chemotherapeutic efficacy by overcoming resistance and alleviating toxicity. Moreover, the indispensable role of microbiota in response to ICIs has been identified in preclinical and clinical studies^77^, ^78^.

### Combination of microbiota‐targeted natural product with chemotherapy

**Figure 2:**
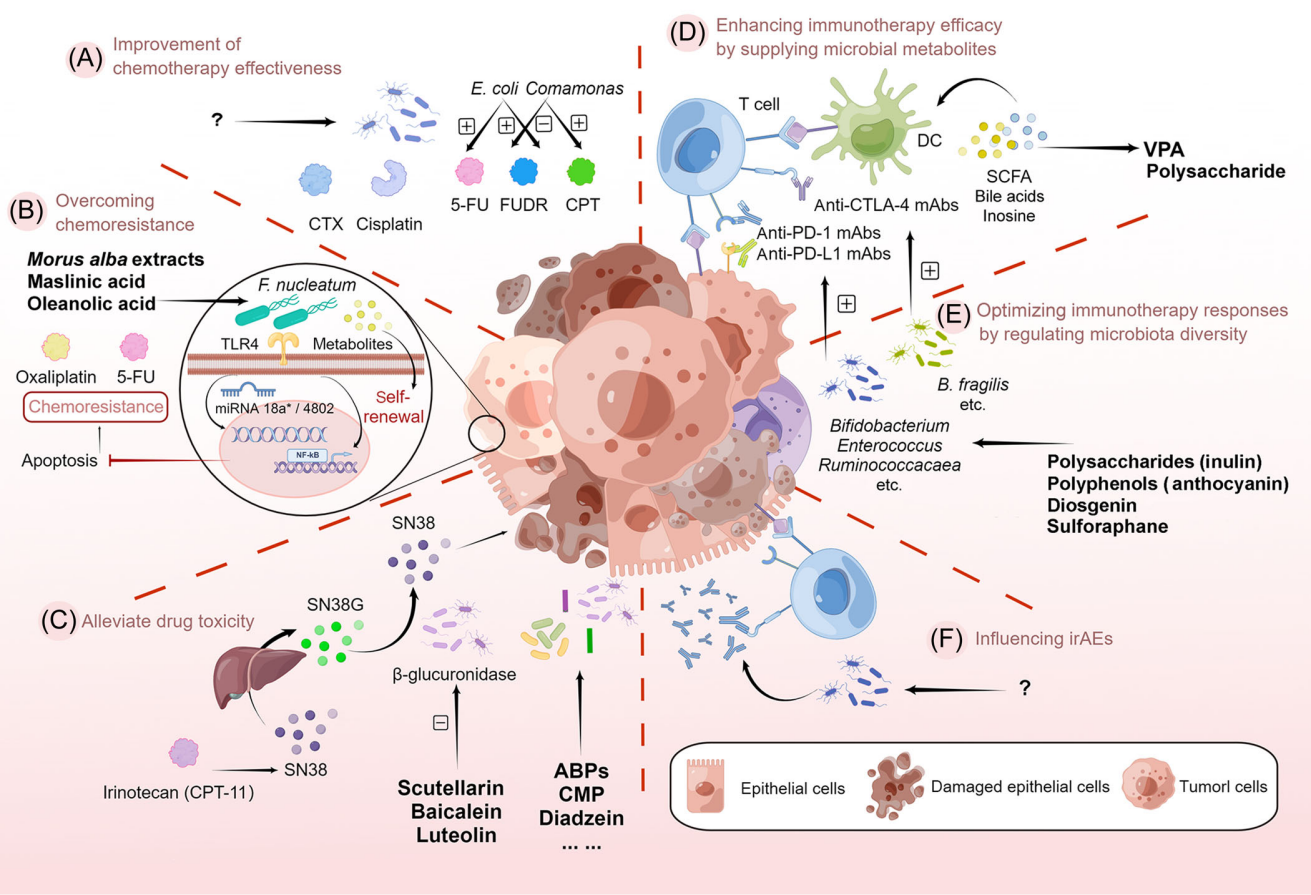
Combination of natural products and therapeutic drugs against cancer by targeting microbiota. (A) The microbiota and microbial metabolites can modulate chemotherapy efficacy. Natural products for enhancing chemosensitivity via microbiota are likely to be found. (B) *Fusobacterium nucleatum* has been found to contribute to chemoresistance. There is a possibility that *Morus alba*, maslinic acid, and oleanolic acid might overcome chemoresistance by targeting *F. nucleatum*. (C) The chemotherapeutic toxicities of irinotecan (CPT‐11) could be alleviated using an *Escherichia coli* β‐glucuronidase inhibitor that includes baicalein, scutellarin, and luteolin. Moreover, natural compounds including ABPs, CMP, and diadzein alleviate 5‐FU‐related toxicity by altering microbial diversity. (D) The immunotherapy efficacy could be enhanced by supplying microbial metabolites, such as VPA and polysaccharides. (E) Natural products regulating microbiota diversity contribute toward optimizing ICI responses. (F) Both specific baseline and dysbiosis are associated with irAEs. More studies are warranted to investigate which and how natural products modulate microbiota to induce immunotherapeutic toxicity. 5‐FU, 5‐Fluorouracil; ABPs, *Albuca bracteate* polysaccharides; CMP, carboxymethyl pachyman; CPT, camptothecin; CTX, cyclophosphamide; FUDR, floxuridine; ICI, immune checkpoint inhibitor irAEs, immune‐related adverse events; SCFA, short‐chain fatty acid; SN38G, SN‐38 glucuronide; TLR‐4, Toll‐like receptor 4; VPA, valproic acid.

Growing evidence has shown that microbiota can modulate chemotherapeutic efficacy. *E. coli* metabolites boosted the efficacy of Floxuridine and 5‐Fluorouracil (5‐FU), whereas *Comamonas* minimized the efficiency of Floxuridine and optimized the effect of Camptothecin^79^, ^80^. The combination of antibiotics targeting Gram‐positive bacteria with cyclophosphamide and cisplatin resulted in a reduced survival time, probably related to microbiota‐induced immune responses^81^, ^82^. The failure of combination therapy is speculatively attributed to the broad‐spectrum antibacterial effects of antibiotics and the dual role of microbiota in tumor immunity. Therefore, more in‐depth analysis of specific microorganisms and their molecular mechanisms is warranted. Of higher practical significance is targeting a specific microbial species at the level of strain, signaling pathway, or effector protein (Figure 2A).

The function of microbiota in chemoresistance is less known and still understudied. To date, *F. nucleatum* has been reported to be associated with the oncogenesis of CRC, drug resistance, and adverse prognostic outcomes. Accumulating studies revealed that *F. nucleatum* becomes a chemoresistant accomplice of 5‐FU and oxaliplatin through preventing apoptosis and promoting cancer stem cell renewal^83^, ^84^. The expression of gene *BIRC3*, which is mediated by microbiome–TLR4–NF‐κB signaling, inhibited caspase‐dependent apoptosis^14^. The transcription of autophagy‐related genes, mediated by miRNA‐18a*/4802, was activated to inhibit apoptosis by microbiome–TLR4–MyD88 signaling^85^. Conversely, tumor cells' self‐renewal and metastasis can be promoted by *F. nucleatum*‐derived metabolites through Notch signaling and Aryl Hydrocarbon Receptor signaling^86^, ^87^. Therefore, *F. nucleatum* is an ideal target for intervention strategies to overcome chemoresistance and improve efficacy.

In this regard, there is a possibility that natural products that supress *F. nucleatum* could delay resistance and attenuate oncogenesis (Figure 2B). *Morus alba* extracts inhibited the reproduction of *F. nucleatum* and *Streptococcus mutans* in an oral environment^88^. Combined with 5‐FU, *Morus alba* extracts enhanced anticancer activity indirectly through an immune response mediated by TLR‐4 signaling^89^. Because of this, *Morus alba* extracts might be promising concomitant drug in antitumor chemotherapy. Furthermore, despite the antibacterial effect, wild blueberry extracts failed to show any complementary function with 5‐FU^90^, ^91^. Similarly, quinoa extracts did not show relevant activities despite the excellent antibacterial activity against *F. nucleatum* ^92^, ^93^. More experiments focused on other natural products targeting *F. nucleatum*, such as maslinic acid from *Olea europaea*, oleanolic acid from *Pistacia lentiscus*, and iodoform‐containing “MGP” gutta‐percha (also called mediated gutta‐percha), are still required to validate the antitumor effect^94^, ^95^. The ineffectiveness may be related to the nonmicrobiota specificity and other biological characteristics of these natural products. Nanomaterial‐loaded M5 phage, which can selectively eliminate *F. nucleatum*, was believed to help potentiate drug bioavailability in mouse models of CRC^96^. *F. nucleatum* vaccines are suggested to have potential in cancer prevention^97^.

The ubiquitous chemotherapeutic toxicities of irinotecan (Camptothecin‐11, also called CPT‐11) and 5‐FU, involving diarrhea and intestinal mucositis, are regulated by gut microbiota^98^, ^99^. SN‐38, the active metabolite of irinotecan, undergoes hepatic decomposition and is subsequently re‐activated by bacterial β­‐glucuronidases in the gut^100^. Meanwhile, CPT‐11 might induce the enrichment of *Enterobacterium* and *Clostridium* to stimulate the activity of β­‐glucuronidases, exacerbating the adverse effects^101^. A specific inhibitor of β­‐glucuronidases can potentially mitigate the side effects and increase tolerance to higher doses of CPT‐11 in CRC patients^102^, ^103^. So far, it has been demonstrated that certain flavonoids, including baicalein, scutellarin, luteolin, and quercetin show strong inhibitory effects against *E. coli* β‐­glucuronidases^104^. Total flavonoids of *Glycyrrhiza uralensis* alleviate irinotecan‐induced colitis via microbiota and associated metabolism^105^. Additionally, recent studies suggested that amentoflavone, a natural extract from *Ginkgo biloba*, was a powerful broad‐spectrum blocker against bacterial β­‐glucuronidases and might be exploited as a promising lead compound for developing new medicines to address intestinal toxicity^106^ (Figure 2C).

Given the unclear mechanism of microbiota–host interaction, there is an urgent need to investigate how natural products could alter microbial diversity to explore the potential of reducing negative effects (especially 5‐FU‐related toxicity). Given that the multilayered flora play a role of multikingdom interactions in chemotherapy, natural compounds altering microbial diversity provide broader application than those targeting single bacterial species. Carboxymethyl pachyman, combined with 5‐FU, relieves intestinal damage and prevents dysbiosis by increasing the proportion of *Lactobacilli, Bacteroidetes*, and bacteria‐produced butyrate^107^. *Albuca bracteate* polysaccharides, combined with 5‐FU, can induce significantly abundant *Anaerostipes, Ruminococcus*, and *Oscillospira*, leading to the synergistic antitumor effect in CT‐26 tumor‐bearing mice^108^. Also, many other natural products, including diadzein, dihydrotanshinone, and patchouli alcohol, exert similar effects^109^ (Figure 2C).

### Combination of microbiota‐targeted natural products with immunotherapy

One of the mechanisms by which microbiota contributes to antitumor immunity and immunotherapy effects is through metabolites. Myriads of small molecules are produced or transformed by microbiota and disseminated to affect the TIME. Specific colonic anaerobes ferment dietary fiber and resistant starch to produce SCFAs. SCFAs play a vital role in the synergy with ICIs by inducing tumor cell apoptosis and promoting CD8^+^ T cell activity^110^. Secondary bile acid, converted from primary bile acids by *Clostridium scindens*, has shown immune‐suppressive effects^111^, ^112^. In contrast, anacardic acid might contribute to ICI responses by activating proven immunity and modulating adaptive immunity^113^, ^114^. Inosine, derived from *Akkermansia muciniphila* and *Bifidobacterium pseudolongum*, has been proven to be essential for ICIs as the energy source of CD8^+^ T cells and Th1^115^. Other metabolites, involving tryptophan, peptidoglycan, and polysaccharide, might play a role in ICIs by influencing the TIME^116^, ^117^. Accordingly, compounds derived by modifying the beneficial metabolites could enhance immunotherapy efficacy, which is expected to be more practical and precise than live bacterial transplants. The SCFA valproic acid (VPA), a class of microbiota‐derived metabolite, has been used in clinical trials in combination with ICIs in patients with entity carcinoma^118^. The exo‐polysaccharide from *Lactobacillus* could increase CCR6^+^ CD8^+^ T cells and modulate the TME to enhance ICI effects^119^. Most recently, it was found that certain enterococci species secrete the NlpC/p60 peptidoglycan hydrolase secreted antigen A (SagA) and produce an immunologically active muropeptide to activate the NOD2 receptor, which enhances anti‐PD‐L1 responses and ICI efficacy^116^ (Figure 2D).

The diversity of commensal microbiota (α‐diversity) modulates partial and general immune responses, contributing to different immunotherapeutic responses. In addition, the extensive microbial variation across adult individuals (β‐diversity) is another factor resulting in heterogeneous responses to ICI therapy. It was discovered that *Bifidobacterium, Enterococcus, Faecalibacterium*, and *Ruminococcaceae* facilitated PD‐1 and PD‐L1 inhibitors against melanoma^77^, ^120^, ^121^, ^122^, ^123^. The anti‐CTLA4 mAbs were assisted by *Bacteroides fragilis* and *Faecalibacterium* in melanoma^78^, ^124^. *A. muciniphila* has been shown to enhance the effectiveness of PD‐1‐ and CTLA‐4‐based immunotherapy against epithelial tumors in nonsmall cell lung cancer, renal cell carcinoma, and urothelial carcinoma^125^.

In addition, *A. muciniphila* and three other species contribute to PD‐L1 and CTLA‐4 inhibition in treating intestinal cancer, bladder cancer, and melanoma^126^. The abundance of critical bacterial species might determine the responsiveness of patients to ICIs and the efficacy of ICIs. Considering the role that microorganisms play in response to ICIs, this is an intriguing topic with the potential for translation. Inulin and inulin gel have been reported to increase the relative abundance of *Akkermansia* and the fraction of *Roseburia* when coupled with anti‐PD‐1 mAbs. The combined strategy can reduce the burden of CRC by elevating both the beneficial commensal microbiota and the number of Tcf1^+^ PD‐1^+^ CD8^+^ T cells^127^.

Anthocyanins, belonging to flavonoids, exerted synergistic effects with anti‐PD‐L1 Abs by modulating intestinal microbiota and enhancing intratumoral CD8^+^ T cells^128^, ^129^. Castalagin, an active compound of polyphenol‐enriched *Camu camu*, could increase *Alistipes* and *Ruminococcaceae* as well as enhance the CD8^+^/FoxP3^+^ CD4^+^ ratio to increase the response when combined with anti‐PD‐1 mAbs^130^. Additionally, considering the impact of specific microbial species alterations on ICIs, the potential exists for some natural compounds to mediate immunotherapy (Figure 2E and Table 1).

**Table 1:** Natural products for regulating microbiota diversity and optimizing immune checkpoint inhibitor responses.

Natural products	Regulation of microbiota diversity	Improvement of TME	Altered metabolites	Animal models	Concomitant application/translational potential
Inulin	*Akkermansia, Lactobacillus* and *Roseburia* ↑			BALB/c mice with CT26 cells^127^	Optimize anti‐PD‐1 Abs responsiveness
Inulin gel	*Akkermansia* and *Roseburia* ↑	Tcf1^+^ PD‐1^+^ CD8^+^ T cells ↑	SCFA ↑		
Anthocyanins	*Lachnospiraceae, Ruminococcaceae, Clostridia* and *Lactobacillus johnsonii* ↑	CD8^+^ T cells ↑		CRC mice^128^, ^129^	Optimize anti‐PD‐L1 Abs responsiveness
Castalagin	*Ruminococcaceae* and *Alistipes* ↑	CD8^+^/FoxP3^+^ CD4^+^ ratio ↑		C57BL/6 J mice^130^	Optimize anti‐PD‐1 Abs responsiveness
Diosgenin	*Lactobacillus* and *Sutterella* ↑, *Bacteroides* ↓	CD4^+^ and CD8^+^ T cells ↑		C57BL/6 mice^131^	Optimize anti‐PD‐1 Abs responsiveness
Ginsenosides	*Bifidobacterium, Lactobacillus, Bacteroides acidifaciens*, and *Bacteroides xylanisolvens* ↑			CRC mice^132^, ^133^	Has the potential to optimize ICI responsiveness
Sulforaphane	*Bacteroides fragilis* and *Clostridium cluster I* ↑			C57BL/6 J mice^134^, ^135^	Has the potential to optimize ICI responsiveness to BC

↑, increase in the mouse model compared with healthy controls; ↓, decrease in the mouse model compared with healthy controls; BC, bladder cancer; CRC, colorectal cancer; ICI, immune checkpoint inhibitor; SCFA, short‐chain fatty acid; Tcf1^+^ PD‐1^+^ CD8^+^ T cells, stem‐like T‐cell factor‐1^+^ PD‐1^+^ CD8^+^ T cells; TME, tumor microenvironment.

ICI treatment eliminates tumor cells while disrupting the equilibrium of the host immune system. There is mounting evidence that microbiota have a dual‐edged function in immune‐related adverse events (irAEs). Studies to date demonstrated that both specific baseline and dysbiosis were associated with ICIs‐induced toxicity^136^. Under such conditions, exploring how natural products alter diversity and counter dysbiosis could hold translational promise for immunotherapeutic efficacy by minimizing toxicity (Figure 2F).

### New targeted therapy by genetically engineered microbiota

Recently, targeted enzymes or bioactive compounds have been synthesized using modified microbes. Researchers used chassis cells to express functional genes or gene clusters in a heterogeneous manner to address diseases such as inflammatory bowel disease and CRC^137^, ^138^. However, the persistent expression of foreign genes in chassis cells using synthetic biology may lead to increased metabolic burden and unanticipated adverse effects. To provide more accurate and effective treatment results, scientists have produced “smart” bacteria that can detect and react to microenvironmental signals^139^, ^140^. The application of live bacteria and engineered strains implies that retaining microbial receptors and immunogenicity is more conducive to the targeted therapy of prebiotics and natural products.

Collectively, the interplay between microbiota and natural products in cancer therapy is evolving toward greater individual precision and targeted precision. However, microbiota‐directed anticancer therapies are confronted with challenges in terms of safety. Microbiota presents new targets for natural product therapies, but the combination of drugs used needs to be approached with caution. In recent studies, drug combinations have been subjected to systematic evaluation via the Genomics of Drug Sensitivity in Cancer (GDSC) cell line screening platform, which provides prospects for potential clinical application^141^.

On the other hand, while synthetic biology has driven the rapid development of engineered bacteria, utilizing live nonmodel bacteria to produce bioactive metabolites poses more significant safety risks than directly supplementing prebiotics. Fecal microbiota transplantation and oral probiotics have proven challenging for colonization and carry infection risks^142^. Therefore, the development of semiviable engineered bacteria—with metabolic activity but without toxicity—might be a promising avenue for utilizing microbiota‐produced natural products in cancer therapy.

## CONCLUDING REMARKS

The commensal microbiota is increasingly believed to play an essential role in cancer development and treatment as a predictive biomarker or prognostic target. Natural products—the endogenous chemical compounds or metabolites of plants, animals, and microorganisms—have drawn considerable attention for their possible therapeutic value and low inherent toxicity. Despite the exciting prospects of natural compounds targeting microbiota to prevent tumor initiation and progression, future investigations remain challenging.

As previously discussed, putative microbiota engages in oncogenesis through genetic damage, epigenetic modulation, signaling cascades, inflammation, and immunity. Yet, the uncovered molecular mechanisms are just the tip of the iceberg of the multilayered pathogenesis. Additional research should focus on multikingdom microbial analyses, such as fungi–bacteria interactions, rather than only bacterial research. In addition, the variable distribution of intratumoral microorganisms and their interactions with distinct cell types is overlooked due to the absence of information on spatial heterogeneity. This is another neglected subject that arises from substantial research. Development of newer microbe–microbe and microbe–host interactions will be facilitated by applying multiomics at the single‐cell level.

There is a need to delve into explicit causation rather than mere correlations between microbiota alterations induced by natural products and their antitumor effects at the level of individual differences. Considering the significant differences in the gut microbiota between mice and humans, more clinical research is warranted to confirm the effects of natural products on human microbiota. Moreover, Big Data analysis offers hope for advancing precision medicine and reducing treatment side effects arising from individual differences.

By targeting multiple molecules and pathways, natural products are undeniably potential candidates for combination therapy to improve antitumor therapeutic effectiveness. Significant challenges in combination therapy for cancer include efficacy and safety. The systematic assessment of combination therapy and advancements in synthetic biology offer promising prospects for potential clinical applications.

Efforts to manipulate the microbiome using natural items to prevent and treat cancer are gaining momentum. The numerous benefits of natural products and the growing significance of microbiota in malignancies herald countless investigation opportunities to inform and create new guidelines for anticancer clinical practices.
